# Exome Sequencing in BRCA1-2 Candidate Familias: The Contribution of Other Cancer Susceptibility Genes

**DOI:** 10.3389/fonc.2021.649435

**Published:** 2021-05-07

**Authors:** Gabriella Doddato, Floriana Valentino, Annarita Giliberti, Filomena Tiziana Papa, Rossella Tita, Lucia Pia Bruno, Sara Resciniti, Chiara Fallerini, Elisa Benetti, Maria Palmieri, Maria Antonietta Mencarelli, Alessandra Fabbiani, Mirella Bruttini, Alfredo Orrico, Margherita Baldassarri, Francesca Fava, Diego Lopergolo, Caterina Lo Rizzo, Vittoria Lamacchia, Sara Mannucci, Anna Maria Pinto, Aurora Currò, Virginia Mancini, Oncologic Multidisciplinary Team, Azienda Ospedaliera Universitaria Senese, Alessandro Neri, Oncologic Multidisciplinary Team, Azienda Usl Toscana Sud Est, Angelo Martignetti, Francesca Mari, Alessandra Renieri, Francesca Ariani

**Affiliations:** ^1^Medical Genetics, University of Siena, Siena, Italy; ^2^Med Biotech Hub and Competence Center, Department of Medical Biotechnologies, University of Siena, Siena, Italy; ^3^Genetica Medica, Azienda Ospedaliera Universitaria Senese, Siena, Italy; ^4^Molecular Diagnosis and Characterization of Pathogenic Mechanisms of Rare Genetic Diseases, Azienda Ospedaliera Universitaria Senese and Clinical Genetics, ASL Toscana SudEst. Ospedale della Misericordia, Grosseto, Italy; ^5^Unit of Pathology, Department of Medical Biotechnology, University of Siena, Siena, Italy

**Keywords:** *BRCA1*, *BRCA2*, cancer susceptibility genes, HBOC, ES (Exome Sequencing)

## Abstract

Hereditary Breast and Ovarian Cancer (HBOC) syndrome is a condition in which the risk of breast and ovarian cancer is higher than in the general population. The prevalent pathogenesis is attributable to inactivating variants of the *BRCA1-2* highly penetrant genes, however, other cancer susceptibility genes may also be involved. By Exome Sequencing (ES) we analyzed a series of 200 individuals selected for genetic testing in *BRCA1-2* genes according to the updated National Comprehensive Cancer Network (NCCN) guidelines. Analysis by MLPA was performed to detect large *BRCA1-2* deletions/duplications. Focusing on *BRCA1-2* genes, data analysis identified 11 cases with pathogenic variants (4 in *BRCA1* and 7 in *BRCA1-2*) and 12 with uncertain variants (7 in *BRCA1* and 5 in *BRCA2*). Only one case was found with a large *BRCA1* deletion. Exome analysis allowed to characterize pathogenic variants in 21 additional genes: 10 genes more traditionally associated to breast and ovarian cancer (*ATM*, *BRIP1*, *CDH1*, *PALB2*, *PTEN*, *RAD51C*, and *TP53*) (5% diagnostic yield) and 11 in candidate cancer susceptibility genes (*DPYD*, *ERBB3*, *ERCC2*, *MUTYH*, *NQO2*, *NTHL1*, *PARK2*, *RAD54L*, and *RNASEL*). In conclusion, this study allowed a personalized risk assessment and clinical surveillance in an increased number of HBOC families and to broaden the spectrum of causative variants also to candidate “non-canonical” genes.

## Introduction

Breast cancer (BC) represents a priority public health problem being the most common cancer in women (1, 2). Families with a history of multiple BC or ovarian cancer (OC) approximately account for 15% of all patients with BC (3). Hereditary breast and ovarian cancer (HBOC) is an adult-onset condition associated with a high risk of breast and ovarian cancer and an increased risk of other cancers such as prostate cancer, pancreatic cancer, and melanoma (4, 5). Since 1994, when *BRCA1* (MIM#113705) and *BRCA2* (MIM#600185) were identified, pathogenic variants (PVs) in these two genes have been known to be the most important cause of HBOC (6–8). In the majority of cases, the *BRCA1* or *BRCA2* PVs are inherited from one of the parents accompanied by a cancer susceptibility transmitted as an autosomal dominant trait. On average, the risk of cancer differs between the two genes, as for *BRCA1* the assessed average risk of breast and ovarian cancers ranges from 57 to 65% and from 20 to 50%, respectively, and for *BRCA2* the risk ranges from 35 to 57% and from 5 to 23%, respectively (9, 10).

The identification of PVs in *BRCA1* or *BRCA2* genes has important clinical implications, as, when detected, represents important information to guide patients therapeutic and preventive choices. Interventions, such as risk-reducing bilateral mastectomy and salpingo-oophorectomy or annual breast magnetic resonance imaging (MRI) screening are available to subjects who carry *BRCA1* or *BRCA2* PVs to enable tumor early detection and active risk reduction (11, 12). The presence of *BRCA1-2* PVs can also impact cancer treatment decisions, principally with regard to the employment of platinum agents or poly(ADP-ribose) polymerase (PARP) inhibitors (13).

It is now estimated that more than one-half of individuals who meet the National Comprehensive Cancer Network (NCCN) testing criteria for HBOC carry PVs in genes other than *BRCA1* or *BRCA2*. Most of these genes encode proteins sharing the same homologous recombination DNA repair function with *BRCA1-2 ATM*, *BRIP1*, *CHEK2*, *NBN*, *PALB2*, *RAD50*, *RAD51C*, and *RAD51D* (14–22). In addition, PVs in genes involved in the overlapping Fanconi anemia (FA) pathway and in the mismatch repair (MMR) pathway have been also found in BC and OC patients (23). Finally, several other genes beyond *BRCA1-2*, often with a not yet fully cleared clinical significance, have been found to be mutated and involved in HBOC cases by exome sequencing (ES) studies over the last years (24, 25).

The objective of the current study was to analyze by ES a series of 200 individuals who meet the National Comprehensive Cancer Network (NCCN) testing criteria for HBOC. This study allowed to go further beyond *BRCA1-2* genes and identify in additional 21 patients with PVs in canonical (*ATM*, *BRIP1*, *CDH1*, *PALB2*, *PTEN*, *RAD51C*, and *TP53*) and candidate non-canonical (*DPYD*, *ERBB3*, *ERCC2*, *MUTYH*, *NQO2*, *NTHL1*, *PARK2*, *RAD54L*, and *RNASEL*) HBOC genes.

## Materials and Methods

### Selection of Patients and DNA Samples’ Preparation

Two hundred patients were selected at the Medical Genetics Unit (A.O.U.S, Siena, Italy) between 2019 and 2020 for Exome Sequencing (ES) according to the updated National Comprehensive Cancer Network (NCCN) guidelines (26). The criteria used to access the test are: breast cancer diagnosed <45 years, breast and ovarian cancer, triple negative breast cancer diagnosed <60 years, male breast cancer diagnosed at any age, bilateral breast cancer with the first diagnosed <50 years, epithelial ovarian cancer diagnosed at any age, exocrine pancreatic cancer diagnosed at any age, and personal history of breast cancer and one close blood relative with breast cancer <50 or ovarian cancer or pancreatic cancer.

Genetic counseling was carried out to evaluate each patient’s personal and familial cancer history considering not only breast and ovarian cancers but also other types of cancer. All patients gave their written informed consent to the study that was carried out according to the Helsinki declaration.

Genomic DNA was extracted from EDTA peripheral blood samples using MagCore HF16 (Diatech Lab Line, Jesi, Ancona, Italy) according to the manufacturer’s instructions. DNA quantity was estimated using the Qubit 3.0 Fluorometer (Thermo Fisher Scientific, Waltham, MA, USA).

### Exome Sequencing

Sample preparation was performed following the *Nextera Flex for Enrichment* manufacturer protocol. The workflow uses a bead-based transposome complex to tagment genomic DNA, which is a process that fragments DNA and then tags the DNA with adapter sequences in one step. After saturation with input DNA, the bead-based transposome complex fragments a set number of DNA molecules. This fragmentation provides flexibility to use a wide DNA input range to generate normalized libraries of consistent tight fragment size distribution. Following tagmentation, a limited-cycle PCR adds adapter sequences to the ends of a DNA fragment. A subsequent target enrichment workflow is then applied. Following pooling, the double stranded DNA libraries are denatured and biotinylated *TruSight One Expanded Oligonucleotide* probes are hybridized to the denatured library fragments. After hybridization, Streptavidin Magnetic Beads (SMB) then capture the targeted library fragments within the regions of interest. The captured and indexed libraries are eluted from beads and further amplified before sequencing. The exome sequencing analysis was performed on the Illumina *NovaSeq6000 System* (Illumina San Diego, CA, USA) according to the NovaSeq6000 System Guide. Reads were mapped against the hg19 reference genome by using the Burrow-Wheeler aligner BWA (27). Variant calling was obtained using an in-house pipeline which takes advantage of the GATK Best Practices workflow (28).

ES data were filtered using eVai (enGenome) software. Variants’ prioritization was obtained by using increasingly enlarged filters: i) genes (*BRCA1* and *BRCA2*); ii) phenotype (using HPO terms: Breast and ovarian neoplasms); iii) phenotype (using HPO terms: Neoplasms). In order to find PVs we focused our attention on rare variants (minor allele frequency, MAF <0,01). Frameshift, stopgain, and splice site variants were prioritized as pathogenic. Missense variants were predicted to be damaging by CADD-phred prediction tools (score ≥25) and splice site variants by MaxEntScan tool.

The following public databases were used for the interpretation of the variants: ClinVar (https://www.ncbi.nlm.nih.gov/clinvar/), LOVD (https://databases.lovd.nl/shared/genes), the Human Genome Mutation Database (HGMD, http://www.hgmd.cf.ac.uk/ac/index.php), and BRCA Exchange database (https://brcaexchange.org/).

### Sanger Sequencing

Selected variants (VUS and PVs) were confirmed by Sanger sequencing. DNAs were sequenced using the PE Big Dye Terminator Cycle Sequencing Kit on an ABI Prism 3130 analyzer (Applied Biosystems). The data were analyzed using the Sequencher version 4.9 software.

### MLPA

SALSA MLPA (Multiplex Ligation-dependent Probe Amplification) probe mixes P002-D1 and P090-C1 were used for large deletions/duplications analysis in *BRCA1*-*2*, according to the manufacturer’s instructions (MRC-Holland, Amsterdam, Netherlands). MLPA amplicons were run on ABI 3130 Genetic Analyzer (Applied Biosystems, Foster City, USA) while the collected data were analyzed using Coffalyser.net Software (MRC-Holland). Peak heights were normalized and deletions/duplications were defined as recommended by the manufacturer.

### RNA Analysis

Total RNA was isolated from PAXgene blood RNA tubes (PreAnalytiX^®^, Qiagen, Hilden, Germany) (http://www.qiagen.com), with PAXgene Blood RNA Kit (IVD) (PreAnalytiX^®^), following the manufacturer’s instructions. RNA quality was evaluated by NanoDrop 2000 Spectrophotometer (Thermo Scientific). For each sample, 1 µg of RNA was reverse-transcribed into cDNA using a dedicated Qiagen kit (QuantiTect^®^Reverse Transcription Kit, Qiagen) according to the manufacturer’s instructions. Amplification was conducted on a Thermal Cycler 2720 (Applied Biosystems) using primers: exons 3-4 Fw (5’-TCACTGGTACTCTTTCTAATCC-3’), exon 7 Rv (3’-TTCAGGAGCAAAGCTGATCTG-5’). Altered transcript pattern was confirmed by comparison with the transcript patterns observed in healthy control. Normal and aberrant bands were excised from the agarose gel, purified using the MinElute Gel Extraction Kit (Qiagen). DNAs were sequenced by Sanger sequencing using the PE Big Dye Terminator Cycle Sequencing Kit on an ABI Prism 3130 analyser (Applied Biosystems). The data were analyzed using the Sequencher version 4.9 software.

## Results

In the present study, 200 index patients were selected for analysis of *BRCA1-2* genes according to the updated National Comprehensive Cancer Network (NCCN) guidelines. Exome sequencing (ES) followed by a virtual panel focusing on *BRCA1* and *BRCA2* genes were firstly performed. We obtained a mean coverage depth of 120× for targeted sequenced regions (range, 50–180×). This analysis was flanked by MLPA for the identification of large *BRCA1-2* deletions/duplications. Pathogenic variants (PVs) were filtered according to frequency, mutation category, literature, and mutation database data (*BRCA* exchange, ClinVar database). After *BRCA1-2* analysis, PVs in other cancer susceptibility genes were searched and classified according to whether they were traditionally associated (canonical HBOC genes) or not (candidate non-canonical HBOC genes) to breast and ovarian cancer.

### Clinical Features of Cancer Patients

The study cohort included a total 200 index subjects (185 females and 15 males; age 34–95 years) with a clinical suspicion of HBOC on the basis of individual and/or family history. The average age of first cancer diagnosis was 55 years (range: 27–87 years) while the average age of second malignancy diagnosis was 61 years (range: 28–94 years). At first diagnosis 150 index patients had breast cancer (BC), 13 ovarian cancer (OC), 4 prostate cancer (PrC), 17 pancreatic cancer (PC), 2 melanoma (M), 8 other types of cancer, and 6 family history of cancer. Considering BC patients, 122 were monolateral and 23 bilateral cases and 5 unknown. Among bilateral BC, 13 patients had metachronous BC and 10 synchronous BC. The range between the first and the second diagnosis was 1–27 years (**Table 1**). Relating to BC histology, 22 had ductal carcinoma in situ, 78 had invasive ductal carcinoma, 3 had lobular carcinoma *in situ*, 20 invasive lobular carcinoma, and 27 other types of cancer. As regards the second tumor, 3 had ductal carcinoma *in situ*, 13 had invasive ductal carcinoma, 3 invasive lobular carcinoma, and 24 other types of cancer. BC therapy was mastectomy in 40 cases, breast conserving therapy in 78 cases, and unknown in 36 cases (**Table 2**).

**Table 1:** Clinical characteristics of HBOC patients.

HBOC PATIENTS	N°
**SEX**	
Female	185
Male	15
**AGE AT COUNSELING**	**Median (range)**
	34–95
**AGE AT DIAGNOSIS (years)**	**Median (range)**
Primary tumor	27–87
Secondary tumor	28–94
FIRST DIAGNOSIS	**N°**
Breast Cancer	150
Ovarian Cancer	13
Prostate Cancer	4
Pancreatic Cancer	17
Melanoma	2
Others	8
Family history	6
**TIME BETWEEN 1st and 2nd tumors (years)**	1–27

**Table 2:** Clinical characteristics of Breast Cancer (BC) patients.

BC PATIENTS	N
**TUMOR DESCRIPTION**	
Monolateral	122
Bilateral metachronous	13
Bilateral synchronous	10
Unknown	5
**HISTOLOGY 1st breast cancer**	
Ductal *in situ*	22
Ductal invasive	78
Lobular *in situ*	3
Lobular invasive	20
**OTHER TYPE OF CANCER**	27
**HISTOLOGY 2nd breast cancer**	
Ductal *in situ*	3
Ductal invasive	13
Lobular *in situ*	0
Lobular invasive	3
**OTHER TYPE OF CANCER**	24
**TYPE OF SURGERY**	
Mastectomy	40
Conserving therapy	78
Unknown	36

### BRCA1-2 Variants and Large Deletions/Duplications

*BRCA1/2*-targeted analysis revealed 11 pathogenic variants (4 in *BRCA1* and 7 in *BRCA2*) and 12 Variants of Uncertain significance (VUS) (7 in *BRCA1* and 5 in *BRCA2*) (**Table S1**) (**Table S2**). PVs were two nonsense substitutions, seven frameshift deletions, one splicing, and one missense change (**Table S1**). Among PVs, nine were already reported in literature or in the *BRCA* Exchange database (29–35). Seven out of 11 (63%) variants were located in exon 11. One variant (c.4485-1G>T) was intronic and predicted to impact the splicing of mRNA (MaxEntScan). VUS were mostly missense substitutions located in exon 11 (8/12; 66%) (**Table S2**). Seven VUS were already reported in ClinVar database and five were new (**Table S2**). MLPA analysis for *BRCA1-2* of the same samples revealed only one rearrangement (1/200; 0,5%): a deletion on exon 20 of the *BRCA1* gene (rsa 17q21(exon 20)x1) (**Figure S1**). Clinical features of patients with *BRCA1-2* aberrations are reported in **Figure S2**.

### Pathogenic Variants in Canonical HBOC Genes

Beyond *BRCA1-2* genes, ES data analysis revealed 10 pathogenic variants in 7 HBOC-related genes (canonical genes): *ATM*, *BRIP1*, *CDH1*, *PALB2*, *PTEN*, *RAD51C*, and *TP53*. Among these variants, six were nonsense, three were missense, and one splicing variant. Eight variants were already reported in literature or in ClinVar database (**Table 3**) (36–43).

**Table 3:** Pathogenic variants found in canonical HBOC genes.

Patient ID	Gene	Transcript (hg19)	Location(Exon/Intron)	Variant (HGVS)	Protein(HGVS)	MAF (gnomAD%)	dpSNP	ClinVar Classification	CADD	MaxEntScan	Reference
1959/20	*ATM*	NM_000051.3	9	c.1102C>T	p.(Gln368*)	NR	NR	NR	NA	NA	NR
977/20	*BRIP1*	NM_032043.2	17	c.2392C>T	p.(Arg798*)	0.015	rs137852986	Pathogenic​	NA	NA	Wang et al. (36)
2688/20	*CDH1*	NM_004360.5	6	c.781G>T	p.(Glu261*)	0.0012	rs121964873	Likely pathogenic	NA	NA	Berx et al. (37)
434/20	*PALB2*	NM_024675.3	5	c.2257C>T	p.(Arg753*)	0.0024	rs180177110	Pathogenic​	NA	NA	Vagena et al. (38)
589/20	*PALB2*	NM_024675.3	5	c.2336C>G	p.(Ser779*)	0.0012	rs764509489	Pathogenic​	NA	NA	NR
942/20	*PTEN*	NM_000314.7	5	c.277C>T	p.(His93Tyr)	NR	rs786204927	Likely pathogenic	25.9	NA	Ngeow et al. (39)
2761/19	*PTEN*	NM_000314.7	5	c.334C>G	p.(Leu112Val)	NR	NR	NR	29.7	NA	Reifenberger et al. (40)
2834/20	*RAD51C*	NM_058216.1	4	c.577C>T	p.(Arg193*)	0.004	rs200293302	Pathogenic	NA	NA	Song et al. (41)
2070/20	*TP53*	NM_000546.4	5	c.560-1G>A	NA	0.003	rs1202793339	Pathogenic	NA	Decrease the recognition of the splice acceptor site	Dutta et al. (42)
1165/20	*TP53*	NM_000546.4	8	c.841G>A	p.(Asp281Asn)	0.0004	rs764146326	Pathogenic​	31	NA	De Andreade et al. (43)

HGVS, Human Genome Variation Society (http://www.hgvs.org); ClinVar, Clinical Variation database (https://www.ncbi.nlm.nih.gov/clinvar/); MAF, Minor Allele Frequency; CADD, combined annotation dependent depletion; NA, non-applicable; NR, non-report. *means change in a stop codon.

**Figure 1:**
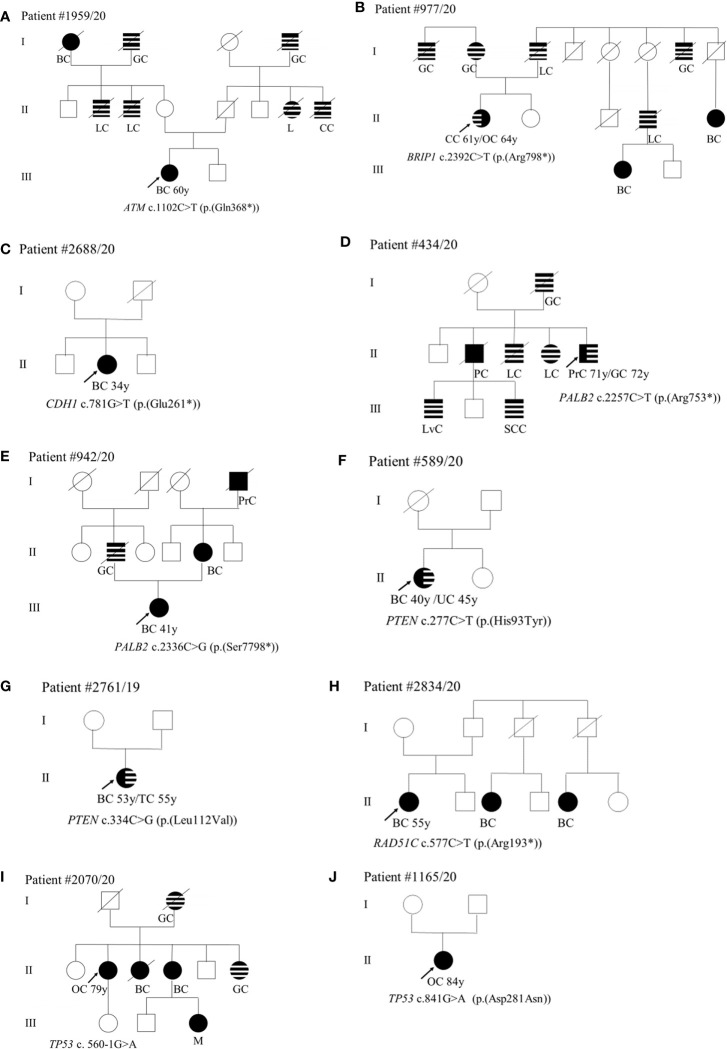
**(A–J)** Pedigrees of patients carrying pathogenic variants in canonical genes. The gene and their variants are reported below the symbol of each proband. The arrows indicate the proband from each family. The black symbols indicate “HBOC-spectrum phenotypes” while striped symbols indicate “other neoplasms.” AC, abdominal cancer; BC, breast cancer; CC, colorectal cancer; GC, gastric cancer; GIC, gastrointestinal cancer; KC, kidney cancer; L, leukemia; LC, lung cancer; LvC, liver cancer; M, melanoma; OC, ovarian cancer; PC, pancreatic cancer; PrC, prostate cancer, S, sarcoma; SCC, spinal cord cancer; TC, tongue cancer; TyC, Thyroid cancer; UC, uterine cancer; X, not defined.

With regard to the clinical phenotype, the index patient (III-1) with the PV in the *ATM* gene (c.1102C>T; p.(Gln368*)) was diagnosed with bilateral BC at 60 years (**Figure 1A**). Histological analysis indicated multifocal infiltrating ductal carcinoma. As concerns the familial affected individuals, two maternal uncles (II-2 and II-3) resulted to be affected with lung cancer diagnosed at 70 years and 50 years, respectively, a paternal aunt with leukemia (II-7) and a paternal uncle (II-8) with colorectal cancer (**Figure 1A**). Individual (I-1) had BC at 80 years and the two grandfathers (I-2 and I-4) died of gastric cancer (**Figure 1A**). The patient (II-1) with the *BRIP1* PV (c.2392C>T; p.(Arg798*)) developed colorectal adenocarcinoma at 61 years and ovarian serous carcinoma at 64 years. The mother (I-2) developed gastric cancer at 65 years and her brother (I-1) died of gastric cancer at 65 years. The father (I-3) was diagnosed with lung cancer at 64 years and, among paternal relatives, there are two cases with BC at 46 years (III-1) and at 44 years (II-5), a case with lung cancer (II-4) and another case with gastric cancer at 72 years (I-7) (**Figure 1B**). The patient with the (c.781G>T; p.(Glu261*)) variant in *CDH1* had bilateral lobular BC at 34 years (**Figure 1C**). The patient (II-5) with *PALB2* PV (c.2257C>T; p.(Arg753*)) was affected by prostate cancer at 71 years and by gastric cancer at 72 years. He had one sister (II-4) and one brother (II-3) with lung cancer (both at 70 years), another brother (II-2) with pancreatic cancer (70 years), a nephew (III-1) with liver cancer, and another nephew (III-3) with spinal cord cancer. The father (I-2) died of gastric cancer at 30 years (**Figure 1D**). The case (III-1) with the (c.2336C>G; p.(Ser7798*)) PV in *PALB2* was diagnosed with unilateral infiltrating ductal BC at the age of 41 years (**Figure 1E**). The father had gastric cancer (II-2), the mother developed BC at 40 years (II-4), and the maternal grandfather prostate cancer at 80 years (I-4) (**Figure 1E**). Concerning PVs in *PTEN*, the patient (II-1) harboring the (c.277C>T; p.(His93Tyr)) substitution was diagnosed with ductal invasive BC at 40 years. At 45 years she had uterine adenocarcinoma (**Figure 1F**). The case (II-1) with the (c.334C>G; p.(Leu112Val)) variant had a personal history of ductal bilateral BC (53 years) (**Figure 1G**). At the age of 55 years, the patient underwent left partial glossectomy for microinvasive carcinoma of the left lingual edge (**Figure 1G**). The patient (II-1) with the (c.577C>T; p. (Arg193*)) nonsense variant in *RAD51C* had BC at 55 years (**Figure 1H**). Two paternal cousins were affected by BC at the age of 45 (II-3) and 35 (II-5) years, respectively (**Figure 1H**). The two patients (II-1) with *TP53* PVs (c.560-1G>A and c.841G>A; p.(Asp281Asn)) were both diagnosed with ovarian serous carcinoma, one at 79 years (**Figure 1I**) and the other at 84 years (**Figure 1J**). The patient with the c.560-1G>A splicing change has two sisters with a diagnosis of BC (at 62, II-3 and at 70 years, II-4) and a sister (II-6) with gastric cancer (69 years) (**Figure 1I**). The mother (I-2) also had gastric cancer at 63 years and the nephew (III-3) melanoma at 56 years (**Figure 1I**). As concerns the second patient (c.841G>A; p.(Asp281Asn)), no familiarity for cancer was reported (**Figure 1J**).

### Pathogenic Variants in Non-Canonical HBOC Genes

Twelve patients had PVs in genes not traditionally associated with HBOC: *DPYP*, *ERBB3*, *ERCC2*, *MUTYH*, *NQO2*, *NTHL1*, *PARK2*, *RAD54L*, and *RNASEL*. Four variants were nonsense, four missense, three splicing, and one frameshift deletion. Nine variants were previously classified as pathogenic or likely pathogenic on the basis of literature and database data (**Table 4**) (44–49).

**Table 4:** Pathogenic variants found in non-canonical HBOC genes.

Patient ID	Gene	Transcript (hg19)	Location (Exon/Intron)	Variant (HGVS)	Protein(HGVS)	MAF (gnomAD%)	dpSNP	ClinVar Classification	CADD	MaxEntScan	Reference
**4011/19**	*DPYD*	NM_000110.3	15	c.1905+1G>A	NA	0.55	rs3918290	Pathogenic	NA	Abolish canonical donor splice site	Stavraka et al. (44)
**146/20**	*ERBB3*	NM_001982.3	3	c.277G>T	p.(Glu93*)	NA	NR	NR	NA	NA	NR
**327/20**	*ERCC2*	NM_000400.3	22	c.2164C>T	p.(Arg722Trp)	0.003	rs121913026	Pathogenic​	24	NA	Boyle et al. (45)
**3216/20**	*MUTYH*	NM_001128425.1	5	c.421del	p.(Gln141Argfs*5)	NA	rs1553129638	Pathogenic​	NA	NA	NR
**2381/20**	*MUTYH*	NM_001128425.1	7	c.536A>G	p.(Tyr179Cys)	0.15	rs34612342	Pathogenic​	25.9	NA	Rizzolo et al. (46)
**79/20**	*MUTYH*	NM_001128425.1	9	c.733C>T	p.(Arg245Cys)	0.0055	rs200495564	Pathogenic​	25.4	NA	Rizzolo et al. (46)
**3006/20**	*MUTYH*	NM_001128425.1	10	c.933+3A>C	NA	0.0074	rs587780751	Pathogenic​	NA	Abolish canonical donor splice site	Pin et al. (47)
**1035/20**	*NQO2*	NM_000904.4	5	c.418-2A>G	NA	0.0047	rs199706530	NR	NA	Abolish canonical acceptor splice site	NR
**2393/20**	*NTHL1*	NM_02528.5	2	c.268C>T	p.(Gln90*)	0.1417	rs150766139	Pathogenic​	NA	NA	Belhadj et al. (48)
**2805/20**	*PARK2*	NM_004562.2	2	c.125G>C	p.(Arg42Pro)	0.0031	rs368134308	Pathogenic​	28.6	NA	NR
**881/20**	*RAD54L*	NM_001142548.1	10	c.1093C>T	p.(Arg365*)	0.0016	rs559500678	NR	NA	NA	NR
**1334/20**	*RNASEL*	NM_021133.3	2	c.793G>T	p.(Glu265*)	0.35	rs74315364	Likely pathogenic	NA	NA	Rokman et al. (49)

HGVS, Human Genome Variation Society (http://www.hgvs.org); ClinVar, Clinical Variation database (https://www.ncbi.nlm.nih.gov/clinvar/); MAF, Minor Allele Frequency; CADD, Combined Annotation Dependent Depletion; NA, non-applicable; NR, non-reported. *means change in a stop codon.

Comparison of variant frequencies with an internal biobank of 1,022 control samples (age ranging between 34 and 95 years) without any history of tumor and analyzed by ES revealed that variants have frequencies <1% (**Table S4**).

**Figure 2:**
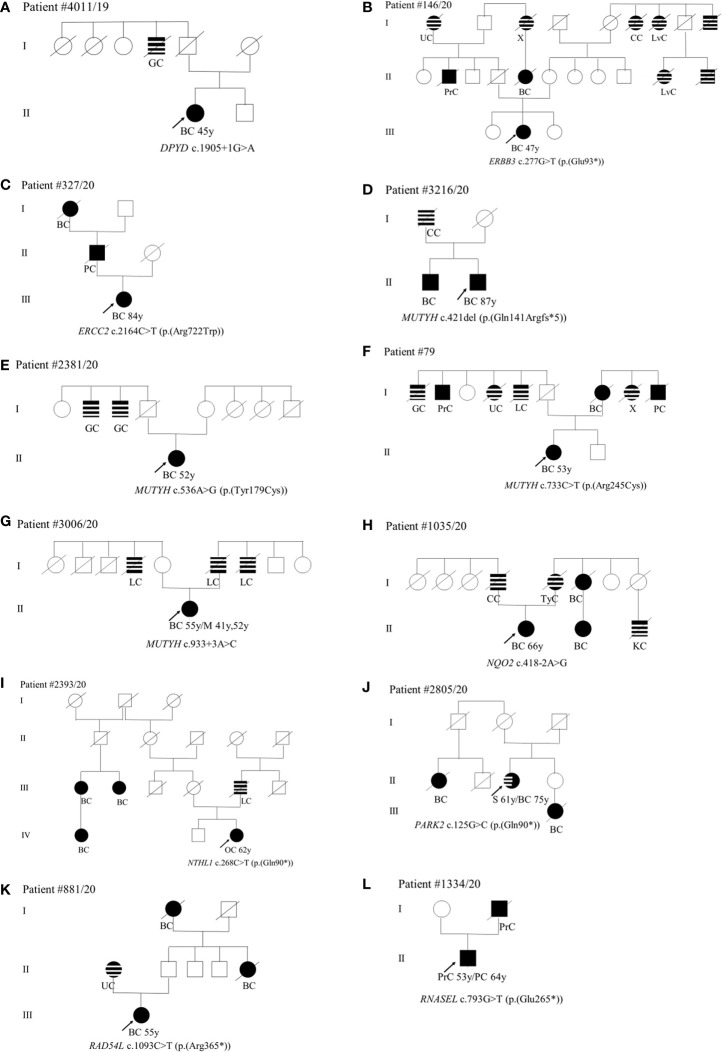
**(A–L)** Pedigrees of patients carrying pathogenic variants in non-canonical genes. The gene and their variants are reported below the symbol of each proband. The arrows indicate the proband from each family. The black symbols indicate “HBOC-spectrum phenotypes” while striped symbols indicate “other neoplasms.” Abbreviations as in **Figure 1**.

As concerns phenotype, we found the c.1905+1G>A splice site variant in the *DPYP* gene in a patient (II-1) diagnosed with unilateral BC at 45 years (**Figure 2A**). Among paternal relatives, an uncle (I-4) died of gastric cancer at 60 years (**Figure 2A**). The *ERBB3* variant (c.277G>T; p.(Glu93*)) was identified in a patient (III-2) with monolateral infiltrating ductal BC diagnosed at 47 years (**Figure 2B**). As concerns paternal relatives, there was an uncle (II-2) dead at 72 years of prostate cancer, one cousin of the father (II-5) dead at 50 years of BC, and a grandmother (I-1) with heteroplasia of the uterus (**Figure 2B**). As regards maternal relatives, there was familiarity for tumors in grandmother siblings (I-6, I-7, I-9) and in cousins of the mother (II-10 and II-11) (**Figure 2B**). The (c.2164C>T; p.(Arg722Trp)) variant in the *ERCC2* gene was found in a patient (III-1) with monolateral infiltrating ductal carcinoma diagnosed at 84 years. The father (II-1) died at 70 years of pancreatic cancer and the paternal grandmother (I-1) died at 60 years of BC (**Figure 2C**). Among the four patients with *MUTYH* PVs, the (c.421del; p.(Gln141Argfs*5)) variant was found in a male patient (II-2) who had monolateral ductal infiltrating BC diagnosed at 87 years (**Figure 2D**). His brother (II-1) had bilateral BC at 71 years and his father (I-1) had colorectal cancer at 55 years (**Figure 2D**). The patient (II-1) with the (c.536A>G; p.(Tyr179Cys)) variant in *MUTYH* had angioma of the upper gingival arch at 36 years and monolateral infiltrating BC at 52 years (**Figure 2E**). Familiarity for gastrointestinal tract neoplasms in the paternal branch (I-2 and I-3) was present. As regards the one with the (c.733C>T; p.(Arg245Cys)) variant had monolateral ductal *in situ* BC diagnosed at 53 years (**Figure 2F**). She had a mother (I-7) who died of bilateral BC at the age of 77 years and a strong family history of cancer including gastric (I-1) prostate (I-2), uterus neoplasia (I-4), lung cancer (I-5), and pancreatic cancer (I-9) (**Figure 2F**). The case (II-1) with the *MUTYH* c.933+3A>C variant had monolateral ductal *in situ* BC at 55 years and two previous melanomas, one in the calf at 41 years and one in the back at 52 years. The father (I-6) and two uncles (I-4 and I-7) were diagnosed with lung cancer (**Figure 2G**). The patient (II-1) with the variant c.418-2A>G in the *NQO2* gene had monolateral ductal infiltrating BC at 66 years (**Figure 2H**). In the family, the father (I-4) had colorectal neoplasm, the mother (I-5) thyroid cancer, one maternal aunt (I-6) and her daughter (II-2) BC, and one cousin (II-3) kidney cancer (**Figure 2H**). We assessed the pathogenicity of this unclassified variant in *NQO2* gene by patient’s mRNA analysis. The analysis showed that this variant abolishes canonical acceptor splice site and results in the formation of two aberrant transcripts: the first skipping of exon 6 p.(Val140_Ala173del)) and the second skipping of exons 5 and 6 p(Phe102_Ala173del)) (data not shown). The (c.268C>T; p.(Gln90*)) stop-gain variant in the *NTHL1* gene was identified in a woman (IV-3) with ovarian cancer (62 years) (**Figure 2I**). In the family, three affected relatives with BC at 44 (III-1), 65 (III-2), and 56 (IV-1) years are present (**Figure 2I**). The father (III-5) died of lung cancer at 63 years (**Figure 2I**). The patient (II-3) with the *PARK2* variant (c.125G>C; p.(Arg42Pro)) was diagnosed with monolateral lobular infiltrating BC at 75 years (**Figure 2J**). Previously she was affected by sarcoma of the left leg (61 years) (**Figure 2J**). In the family, she has a nephew (III-1) who died of BC (46 years) and a maternal cousin (II-1) who died of BC at 50 years (**Figure 2J**). The case (III-1) with the (c.1093C>T; p.(Arg365*)) variant in *RAD54L* had a diagnosis of BC at 55 years (**Figure 2K**). The mother (II-1) had uterus carcinoma at 84 years, a paternal aunt (II-5) and the grandmother (I-1) had juvenile BC (**Figure 2K**). Finally, the case (II-1) mutated in the *RNASEL* gene (c.793G>T; p.(Glu265*)) was diagnosed with prostate cancer at 53 years and with pancreatic cancer at 64 years (**Figure 2L**). His father (I-2) died of prostate cancer at 62 years (**Figure 2L**).

### Uncertain Variants in Cancer Genes Beyond BRCA1-2

In the analysis of ES data, 74 variants were classified as VUS (class 3) on the basis of the IARC recommendations (**Table S3**). Most VUS were located in *ATM* and *PALB2* genes (**Table S3**). Fourteen patients carried more than one VUS on different genes. As regards to mutation types, most were missense variants and the remaining were duplications or deletions. Combined Annotation Dependent Depletion (CADD) was considered to predict the effect of the missense variants.

## Discussion

Multigene panel genetic tests are increasingly employed for the screening of patients presenting HBOC (14–22). However, multigene panel allows a limited gene analysis while Exome Sequencing (ES) allows the simultaneous assessment of virtually an unlimited number of genes. Previous studies on ES employed for the analysis of HBOC patients demonstrated that it is a powerful tool for the identification of PVs in known HBOC-related genes (canonical) and for the discovery of novel disease factors (non-canonical) (24, 25). However, ES analysis has the limit of not identifying Copy Number Variants (CNVs) and PVs in intronic regulatory elements. Here, we analyzed by ES a cohort of 200 patients with a diagnosis of HBOC according to the updated NCCN guidelines (26). ES data analysis allowed us to identify 11 cases with pathogenic variants (4 in *BRCA1* and 7 in *BRCA2*) (5,5%) and 12 with uncertain variants (7 in *BRCA1* and 5 in *BRCA2*). Only one case was found with a large *BRCA1* deletion. The relatively low diagnostic yield in *BRCA1-2* genes is probably due to the enlarged updated diagnostic NCCN guidelines. ES analysis allowed to identify PVs in additional 21 individuals: 10 with PVs in genes already associated to breast and ovarian cancer (*ATM*, *BRIP1*, *CDH1*, *PALB2*, *PTEN*, *RAD51C*, and *TP53*) and 11 in other candidate cancer susceptibility genes (*DYPD*, *ERBB3*, *ERCC2*, *MUTYH*, *NTHL1*, *NQO2*, *PARK2*, *RAD54L*, and *RNASEL*).

Regarding canonical HBOC genes, the genes more frequently mutated were *PTEN* (2/200; 1%), *TP53* (2/200; 1%), and *PALB2* (2/200; 1%). It is well known that BC can be included within several other described cancer syndromes, including Li-Fraumeni syndrome and Cowden syndrome. Thus, it is established that women who carry PVs in *TP53* and *PTEN* have an increased risk of BC (50, 51). In accordance, the two patients with PVs in *PTEN* were diagnosed with BC. The two patients mutated in *TP53* were both diagnosed with ovarian serous carcinoma. Loss-of-function mutations in the *PALB2* gene confer a predisposition to BC with a prevalence of approximately 1% (52). We found two cases with *PALB2* PVs, one with unilateral infiltrating ductal BC and a clear maternal cancer familiarity and one with prostate cancer, a personal history of gastric cancer, and familiarity for other neoplasms. The moderate-penetrance gene *ATM*, is mutated in 1–2% of BC, while in our study it has been found mutated only once (1/200) in a patient with multifocal infiltrating ductal carcinoma (53). As concerns *BRIP1*, Cantor et al. firstly reported the gene mutated in early-onset BC patients and subsequently this association was reinforced by Seal and colleagues who reported truncating mutations in *BRIP1* as low-penetrance BC susceptibility alleles (54, 55). Our study reported a nonsense *BRIP1* variant in a patient with ovarian serous carcinoma. In accordance with literature data reporting the presence of *CDH1* PVs in >80% of invasive lobular BC, *CDH1* has been found mutated in a patient with lobular BC (56, 57). Since the discovery of *RAD51C* in 2010 as a gene responsible for hereditary BC and OC, indication of including the gene in routine clinical genetic testing has been controversial, due to the lower prevalence or the absence of mutations found in subsequent studies (58–60). The present study confirms the role of *RAD51C* in HBOC being found mutated in a patient with BC.

Regarding new candidate non-canonical HBOC genes, the most frequently mutated was *MUTYH* (4/200; 2%). The *MUTYH* gene encodes a DNA glycosylase involved in the base excision repair (61). Biallelic *MUTYH* mutations are involved in the pathogenesis of a form of autosomal recessive colorectal adenomatous polyposis (MUTYH-associated polyposis syndrome, MAP) (62). A study focused on *MUTYH*-associated extracolonic cancers demonstrated that monoallelic variants are associated with an increased risk of gastric and liver cancers, as well as a slightly increased risk of endometrial and breast cancers (63). Here, we found four BC patients with *MUTYH* PVs: two with ductal *in situ* and two with ductal infiltrating carcinoma. They all have a strong family history of cancer. The *DPYD* gene encodes a protein that is active in the catabolic pathway of 5-fluorouracil and *DPYD* mutations result in an increased risk of toxicity in cancer patients receiving 5-fluorouracil chemotherapy (64). The rs291593 SNP in *DPYD* is associated with survival of BC patients (65). In our cohort, *DPYP* was mutated in a patient affected by unilateral BC. The variant was already demonstrated to affect the transcript resulting in the skipping of exon 14 leading to an inactive DPYD allele (66). *ERBB3*, encodes for HER3, a member of the EGFR family of receptor tyrosine kinases (67). The role of ERBB3 as somatic driver of tumorigenesis has been well documented, however, there is few data available about the role of germline variants (68). There is only a study reporting the identification of a germline predisposing mutation in a case of erythroleukemia (69). We found an *ERBB3* nonsense variant in a patient with monolateral infiltrating ductal BC and a positive cancer history among both paternal and maternal relatives. *ERCC2* encodes for an essential component of transcription factor IIH involved in basal cellular transcription and nucleotide excision repair (NER) of DNA lesions (36, 70). Data about its involvement in BC are at present not convincing (71). Here, we report an *ERCC2* missense variant in a patient with monolateral infiltrating ductal BC and a positive oncological history. *NQO2* encodes a flavoprotein that catalyzes the 2-electron reduction of various quinones, redox dyes, and the vitamin K menadione (72). In 2009, Yu and colleagues proposed *NQO2* as a susceptibility gene for breast carcinogenesis (73). Here, we identified a patient with a splice site variant in the *NQO2* gene that was never reported. mRNA analysis demonstrated that the variant has splicing impact generating two aberrant transcripts. *NTHL1* initiates DNA base excision repair of oxidized ring saturated pyrimidine residues (74). Biallelic mutations in *NTHL1* are responsible for familial adenomatous polyposis-3 (FAP3) (48). Subsequently, since *NTHL1* mutations can cause a wide variety of cancers in addition to colorectal cancer, the designation “*NTHL1* syndrome” was proposed (75). We found a mutation of this gene in a woman affected by ovarian cancer and subsequently by pancreatic cancer, suggesting an HBOC spectrum phenotype. *PARK2*, encoding a RING domain-containing E3 ubiquitin ligase, was originally identified as a gene responsible for autosomal recessive juvenile Parkinson disease-2 (*PARK2*, or PDJ) (76). In more recent years, *PARK2* has been characterized as a tumor suppressor gene whose loss of heterozygosity (LOH) and loss of expression have been observed in different tumors such as lung, brain, breast, and ovarian cancer (77–79). We found a *PARK2* PV in a patient with sarcoma of the left leg and monolateral lobular infiltrating BC. She has a positive BC family history. *RAD54L* is a gene that was found mutated in primary cancers, including BC (80). In the present study, we identified a BC patient with a truncating variant in *RAD54L*. *RNASEL*, encoding the endoribonuclease RNase L, is a key enzyme in the interferon induced antiviral and anti-proliferate pathway (81, 82). Mutations in *RNASEL* segregate with the disease in prostate cancer families and specific genotypes are associated with an increased risk of prostate cancer (49). In accordance, we found a *RNASEL* stopgain variant in a patient with a diagnosis of prostate cancer and a father dead at 62 years of prostate cancer.

In conclusion, ES analysis in a cohort of HBOC suspected patients allowed a diagnostic yield of a further −5% in non *BRCA1-2* genes. Canonical genes would have been included in a multigene panel for diagnostic purposes while mutations in candidate non-canonical genes would not have emerged if a ES approach had not been carried out, overlapping the boundaries of clinical conditions, often simplistically separated. However, to make a diagnosis other experiments such as segregation analysis should be performed and these genes remains candidates. Variants in *DPYD*, *MUTYH* (c.536A>G and c.933+3A>C), *NTHL1*, and *RNASEL* genes were also found in controls, suggesting that these variants are probably low–penetrance risk alleles. Finally although it is true that this strategy increases the level of complication of the analysis and the number of VUS per sample, their identification appears to be essential for future definitive classification pooling together the data of different studies.

## Supporting Informations

Oncologic Multidisciplinary Team, Azienda Ospedaliera Universitaria Senese, Siena, Italy: Alessandro Neri^5,6^, Donato Casella^6^, Andrea Bernini^6^, Stefania Marsili^7^, Roberto Petrioli^7^, Salvatora Tindara Miano^7^, Alessandra Pascucci^7^, Ignazio Martellucci^7^, Monica Crociani^8^, Marta Vannini^8^, Federica Fantozzi^9^, Andrea Stella^9^, Alessia Carmela Tripodi^9^, Angelamaria Giusti^9^, Alfonso Fausto^9^, Lucia Mantovani^9^, Francesca Belardi^9^

Oncologic Multidisciplinary Team, Azienda Usl Toscana Sud Est: Angelo Martignetti^10^, Amalia Falzetta^10^, Camilla Casi^10^, Paolo Petreni^10^, Beatrice Forzoni^11^, Enrico Tucci^10^, Tiziana Baglioni^10^, Luciana Biscari^10^, Antonia Borgomastro^10^, Pamela Torre^10^, Francesco Di Clemente^10^, Simona Scali^10^, Marianna Turrini^10^, Maria Deligianni^10^, Sabrina Del Buono^11^, Simonetta Magnanini^11^, Alice Baldi^11^, Tommaso Amato^11^, Ulpjana Gjondedaj^11^, Giorgio Bastreghi^10^, Giovanni Angiolucci^11^, Carmelo Bengala^12^, Roberta Di Rocco^12^

^5^ Department of Medicine, Surgery and Neuroscience, University of Siena, Siena, Italy

^6^ Chirurgia Oncologica della mammella, Azienda Ospedaliera Universitaria Senese, Siena, Italy

^7^ Oncologia Medica, Azienda Ospedaliera Universitaria Senese, Siena, Italy

^8^ Radioterapia, Azienda Ospedaliera Universitaria Senese, Siena, Italy

^9^ Senologia, Azienda Ospedaliera Universitaria Senese, Siena, Italy

^10^ Oncology Department, Azienda Usl Toscana Sud Est, Siena, Italy

^11^ Oncology Department, Azienda Usl Toscana Sud Est, Arezzo, Italy

^12^ Division of Medical Oncology, Misericordia Hospital, Azienda USL Toscana Sud Est, Grosseto

## Data Availability Statement

NGS data has been deposited in publicly accessible repositories. The data can be found here: https://www.ncbi.nlm.nih.gov/sra/PRJNA727249.

## Ethics Statement

Ethical review and approval were not required for the study on human participants in accordance with the local legislation and institutional requirements. The patients/participants provided their written informed consent to participate in this study.

## Author Contributions

GD, FV, AR, and FA have made substantial contributions to conceptions and design and have been involved in drafting the paper. AG, FTP, RT, LPB, SR, CF, EB, MP, and MBr have made substantial contributions to acquisition and analysis of the data. MM, AF, AO, MBa, FF, DL, CLR, VL, SM, AP, and AC made clinical evaluation. All authors have given final approval of the version to be published and agree to be accountable for all aspects of the work in ensuring that questions related to the accuracy or integrity of any part of the work are appropriately investigated and resolved. All authors contributed to the article and approved the submitted version.

## Conflict of Interest

The authors declare that the research was conducted in the absence of any commercial or financial relationships that could be construed as a potential conflict of interest.

## Supplementary Material

Supplementary Figure 1 MLPA results for *BRCA1* gene. MLPA results for *BRCA1* gene of a patient (#1369/19) with familial BC. MLPA size probes for each exon (top) and genomic position (bottom), are given on the X axis. Calculated probe ratios of test sample normalized to the reference samples is given on the Y axis. Probe ratios are indicated by the dots. Black dots indicate probes within the confidence interval (ratio 0.7 to 1.3 by default). Red dot reveals a heterozygous deletion, probe ratio 0.5, in the test sample.

Supplementary Figure 2 Pedigrees of patients carrying pathogenic variants in *BRCA1-2*. Pedigree of patients #3027/20, 352/20, 5632/20, 5647/19, 2734/20, 1568/20, 937/20, 1946/20, 2523/20, 1356/20, 1354/20, and 1369/19. The gene and their variants are reported below the symbol of each proband. The arrows indicate the proband from each family. The black symbols indicate “HBOC-spectrum phenotypes” while striped symbols indicate “other neoplasms.” Abbreviations as in **Figure 1**.

Supplementary Table 1 Pathogenic variants found in HBOC genes. HGVS, Human Genome Variation Society (http://www.hgvs.org); ClinVar, Clinical Variation database (https://www.ncbi.nlm.nih.gov/clinvar/); MAF, Minor Allele Frequency; CADD, Combined Annotation Dependent Depletion; NA, non applicable; NR, non reported.

Supplementary Table 2 VUS variants found in HBOC genes. HGVS, Human Genome Variation Society (http://www.hgvs.org); ClinVar, Clinical Variation database (https://www.ncbi.nlm.nih.gov/clinvar/); MAF, Minor Allele Frequency; CADD, Combined Annotation Dependent Depletion; NA, non applicable; NR, non reported.

Supplementary Table 3 VUS found in canonical and non-canonical HBOC genes. HGVS, Human Genome Variation Society (http://www.hgvs.org); ClinVar, Clinical Variation database (https://www.ncbi.nlm.nih.gov/clinvar/); MAF, Minor Allele Frequency; CADD, Combined Annotation Dependent Depletion; NA, non applicable; NR, non reported.

Supplementary Table 4 Comparison of variant frequencies with an internal biobank of 1022 samples (median range 34-95 years) without any history of tumor and analysed by ES. HGVS, Human Genome Variation Society (http://www.hgvs.org).
