# Corrigendum: Exome Sequencing in BRCA1-2 Candidate Familias: The Contribution of Other Cancer Susceptibility Genes

**DOI:** 10.3389/fonc.2021.740860

**Published:** 2021-08-17

**Authors:** Gabriella Doddato, Floriana Valentino, Annarita Giliberti, Filomena Tiziana Papa, Rossella Tita, Lucia Pia Bruno, Sara Resciniti, Chiara Fallerini, Elisa Benetti, Maria Palmieri, Maria Antonietta Mencarelli, Alessandra Fabbiani, Mirella Bruttini, Alfredo Orrico, Margherita Baldassarri, Francesca Fava, Diego Lopergolo, Caterina Lo Rizzo, Vittoria Lamacchia, Sara Mannucci, Anna Maria Pinto, Aurora Currò, Virginia Mancini, Oncologic Multidisciplinary Team, Azienda Ospedaliera Universitaria Senese, Oncologic Multidisciplinary Team, Azienda Usl Toscana Sud Est, Francesca Mari, Alessandra Renieri, Francesca Ariani

**Affiliations:** ^1^Medical Genetics, University of Siena, Siena, Italy; ^2^Med Biotech Hub and Competence Center, Department of Medical Biotechnologies, University of Siena Italy, Siena, Italy; ^3^Genetica Medica, Azienda Ospedaliera Universitaria Senese, Siena, Italy; ^4^Molecular Diagnosis and Characterization of Pathogenic Mechanisms of Rare Genetic Diseases, Azienda Ospedaliera Universitaria Senese and Clinical Genetics, ASL Toscana SudEst. Ospedale della Misericordia, Grosseto, Italy; ^5^Unit of Pathology, Department of Medical Biotechnology, University of Siena, Siena, Italy

**Keywords:** BRCA1, BRCA2, cancer susceptibility genes, HBOC, ES (Exome Sequencing)

In the original article, there was an error. Instead of “Whole Exome Sequencing” we want to change to “Exome Sequencing” throughout the manuscript.

A correction has been made to the ***Title***:

Exome sequencing in *BRCA1-2* candidate familias: the contribution of other cancer susceptibility genes

A correction has been made to the ***Abstract***, ***phrase 3***:

By Exome Sequencing (ES) we analyzed a series of 200 individuals selected for genetic testing in *BRCA1-2* genes according to the updated National Comprehensive Cancer Network (NCCN) guidelines.

A correction has been made to ***Keywords***:

*BRCA1, BRCA2*, cancer susceptibility genes, HBOC, ES (Exome Sequencing)

A correction has been made to ***Introduction***, ***paragraph 3***:

It is now estimated that more than one-half of individuals who meet the National Comprehensive Cancer Network (NCCN) testing criteria for HBOC carry PVs in genes other than *BRCA1* or *BRCA2*. Most of these genes encode proteins sharing the same homologous recombination DNA repair function with *BRCA1-2 ATM, BRIP1, CHEK2, NBN, PALB2, RAD50, RAD51C*, and *RAD51D* (14–22). In addition, PVs in genes involved in the overlapping Fanconi anemia (FA) pathway and in the mismatch repair (MMR) pathway have been also found in BC and OC patients (23). Finally, several other genes beyond BRCA1-2, often with a not yet fully cleared clinical significance, have been found to be mutated and involved in HBOC cases by exome sequencing (ES) studies over the last years (24, 25).

A correction has been made to ***Introduction***, ***paragraph 4***:

The objective of the current study was to analyze by ES a series of 200 individuals who meet the National Comprehensive Cancer Network (NCCN) testing criteria for HBOC. This study allowed to go further beyond *BRCA1-2* genes and identify in additional 21 patients with PVs in canonical (*ATM, BRIP1, CDH1, PALB2, PTEN, RAD51C*, and *TP53*) and candidate non-canonical (*DPYD, ERBB3, ERCC2, MUTYH, NQO2, NTHL1, PARK2, RAD54L*, and *RNASEL*) HBOC genes.

A correction has been made to ***Materials and Methods***:

***Selection of Patients and DNA Samples’ Preparation***,***Paragraph 1***: Two hundred patients were selected at the Medical Genetics Unit (A.O.U.S, Siena, Italy) between 2019 and 2020 for Exome Sequencing (ES) according to the updated National Comprehensive Cancer Network (NCCN) guidelines (26)

EXOME SEQUENCING: ***Exome Sequencing***

***Paragraph 1:***

Sample preparation was performed following the Nextera Flex for Enrichment manufacturer protocol. The workflow uses a bead-based transposome complex to tagment genomic DNA, which is a process that fragments DNA and then tags the DNA with adapter sequences in one step. After saturation with input DNA, the bead-based transposome complex fragments a set number of DNA molecules. This fragmentation provides flexibility to use a wide DNA input range to generate normalized libraries of consistent tight fragment size distribution. Following tagmentation, a limited-cycle PCR adds adapter sequences to the ends of a DNA fragment. A subsequent target enrichment workflow is then applied. Following pooling, the double stranded DNA libraries are denatured and biotinylated TruSight One Expanded Oligonucleotide probes are hybridized to the denatured library fragments. After hybridization, Streptavidin Magnetic Beads (SMB) then capture the targeted library fragments within the regions of interest. The captured and indexed libraries are eluted from beads and further amplified before sequencing. The exome sequencing analysis was performed on the Illumina NovaSeq6000 System (Illumina San Diego, CA, USA) according to the NovaSeq6000 System Guide. Reads were mapped against the hg19 reference genome by using the Burrow-Wheeler aligner BWA (27). Variant calling was obtained using an in-house pipeline which takes advantage of the GATK Best Practices workflow (28).

***Paragraph 2:***

ES data were filtered using eVai (enGenome) software. Variants’ prioritization was obtained by using increasingly enlarged filters: i) genes (*BRCA1* and *BRCA2*); ii) phenotype (using HPO terms: Breast and ovarian neoplasms); iii) phenotype (using HPO terms: Neoplasms). In order to find PVs we focused our attention on rare variants (minor allele frequency, MAF <0,01). Frameshift, stopgain, and splice site variants were prioritized as pathogenic. Missense variants were predicted to be damaging by CADDphred prediction tools (score ≥25) and splice site variants by MaxEntScan tool.

A correction has been made to ***Result***:

***Phrase 2***: Exome sequencing (ES) followed by a virtual panel focusing on *BRCA1* and *BRCA2* genes were firstly performed. We obtained a mean coverage depth of 120× for targeted sequenced regions (range, 50–180×).

A correction has been made to ***Result***:

***Pathogenic Variants in Canonical HBOC Genes***

***Paragraph 1***: Beyond *BRCA1-2* genes, ES data analysis revealed 10 pathogenic variants in 7 HBOC-related genes (canonical genes): *ATM, BRIP1, CDH1, PALB2, PTEN, RAD51C*, and *TP53*. Among these variants, six were nonsense, three were missense, and one splicing variant. Eight variants were already reported in literature or in ClinVar database (Table 3) (36–43).

***Uncertain Variants in Cancer Genes Beyond BRCA1-2***

***Paragraph 1***: In the analysis of ES data, 74 variants were classified as VUS (class 3) on the basis of the IARC recommendations (Table S3). Most VUS were located in *ATM* and *PALB2* genes (Table S3). Fourteen patients carried more than one VUS on different genes. As regards to mutation types, most were missense variants and the remaining were duplications or deletions. Combined Annotation Dependent Depletion (CADD) was considered to predict the effect of the missense variants.

A correction has been made to ***Discussion***:

***Paragraph 1***: Multigene panel genetic tests are increasingly employed for the screening of patients presenting HBOC (14–22). However, multigene panel allows a limited gene analysis while Exome Sequencing (ES) allows the simultaneous assessment of virtually an unlimited number of genes. Previous studies on ES employed for the analysis of HBOC patients demonstrated that it is a powerful tool for the identification of PVs in known HBOC-related genes (canonical) and for the discovery of novel disease factors (non-canonical) (24, 25). However, ES analysis has the limit of not identifying Copy Number Variants (CNVs) and PVs in intronic regulatory elements. Here, we analyzed by ES a cohort of 200 patients with a diagnosis of HBOC according to the updated NCCN guidelines (26). ES data analysis allowed us to identify 11 cases with pathogenic variants (4 in *BRCA1* and 7 in *BRCA2*) (5,5%) and 12 with uncertain variants (7 in *BRCA1* and 5 in *BRCA2*). Only one case was found with a large *BRCA1* deletion. The relatively low diagnostic yield in *BRCA1-2* genes is probably due to the enlarged updated diagnostic NCCN guidelines. ES analysis allowed to identify PVs in additional 21 individuals: 10 with PVs in genes already associated to breast and ovarian cancer (*ATM, BRIP1, CDH1, PALB2, PTEN, RAD51C*, and *TP53*) and 11 in other candidate cancer susceptibility genes (*DYPD, ERBB3, ERCC2, MUTYH, NTHL1, NQO2, PARK2, RAD54L*, and *RNASEL*).

***Paragraph 4***: In conclusion, ES analysis in a cohort of HBOC suspected patients allowed a diagnostic yield of a further −5% in non *BRCA1-2* genes. Canonical genes would have been included in a multigene panel for diagnostic purposes while mutations in candidate non-canonical genes would not have emerged if a ES approach had not been carried out, overlapping the boundaries of clinical conditions, often simplistically separated. However, to make a diagnosis other experiments such as segregation analysis should be performed and these genes remains candidates. Variants in *DPYD, MUTYH* (c.536A>G and c.933+3A>C), *NTHL1*, and *RNASEL* genes were also found in controls, suggesting that these variants are probably low–penetrance risk alleles. Finally although it is true that this strategy increases the level of complication of the analysis and the number of VUS per sample, their identification appears to be essential for future definitive classification pooling together the data of different studies.

The authors apologize for this error and state that this does not change the scientific conclusions of the article in any way. The original article has been updated.

## Publisher’s Note

All claims expressed in this article are solely those of the authors and do not necessarily represent those of their affiliated organizations, or those of the publisher, the editors and the reviewers. Any product that may be evaluated in this article, or claim that may be made by its manufacturer, is not guaranteed or endorsed by the publisher.

