# Supplementary figures and images for: Exome Sequencing in BRCA1-2 Candidate Familias: The Contribution of Other Cancer Susceptibility Genes
Source: Front Oncol. 2021 May 7;11:649435. doi: 10.3389/fonc.2021.649435 (PMC8139251; doi:10.3389/fonc.2021.649435)

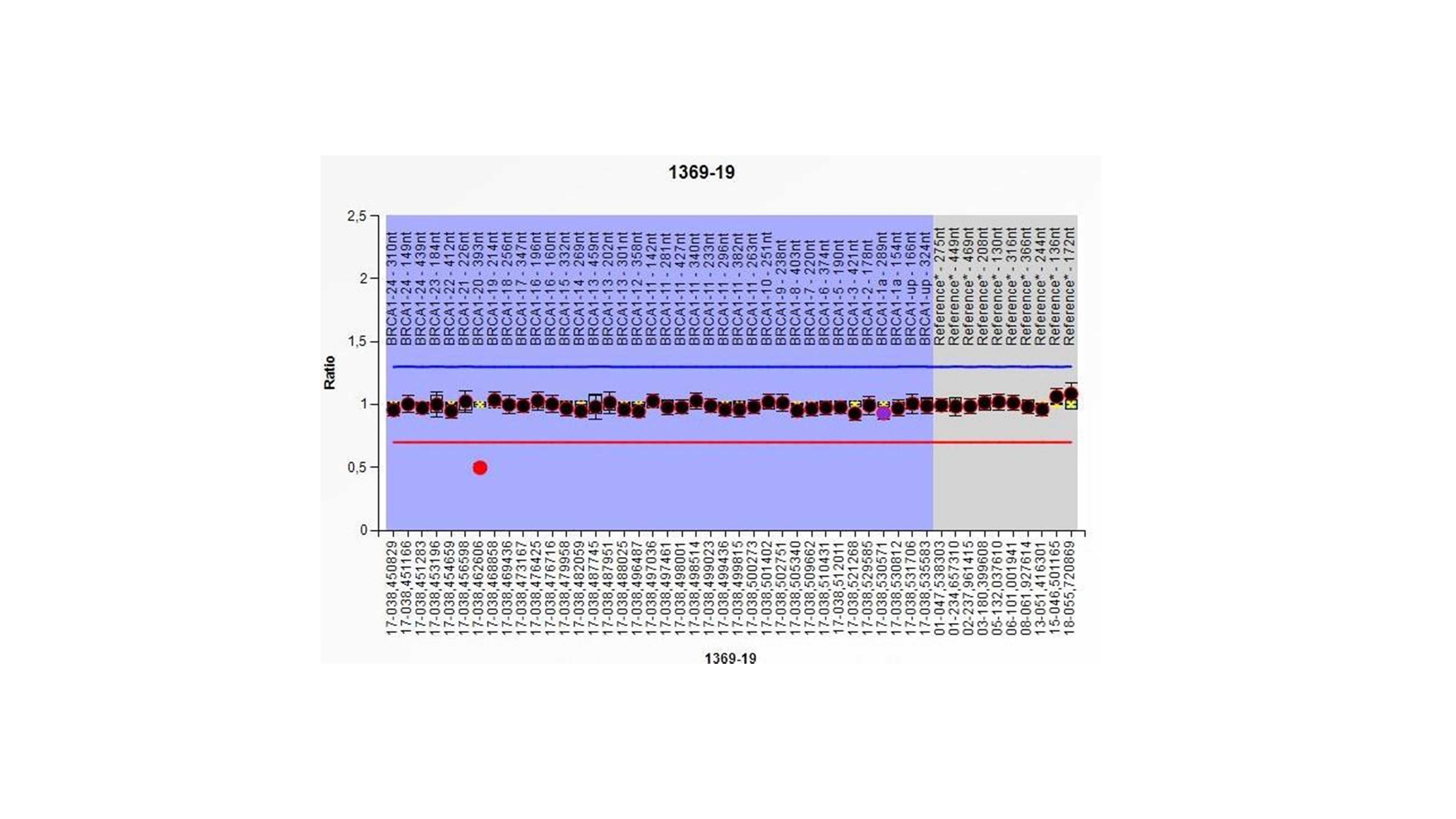

Supplement: Supplementary Figure 1 — MLPA results for BRCA1 gene. MLPA results for BRCA1 gene of a patient (#1369/19) with familial BC. MLPA size probes for each exon (top) and genomic position (bottom), are given on the X axis. Calculated probe ratios of test sample normalized to the reference samples is given on the Y axis. Probe ratios are indicated by the dots. Black dots indicate probes within the confidence interval (ratio 0.7 to 1.3 by default). Red dot reveals a heterozygous deletion, probe ratio 0.5, in the test sample. [file Image_1.jpeg]

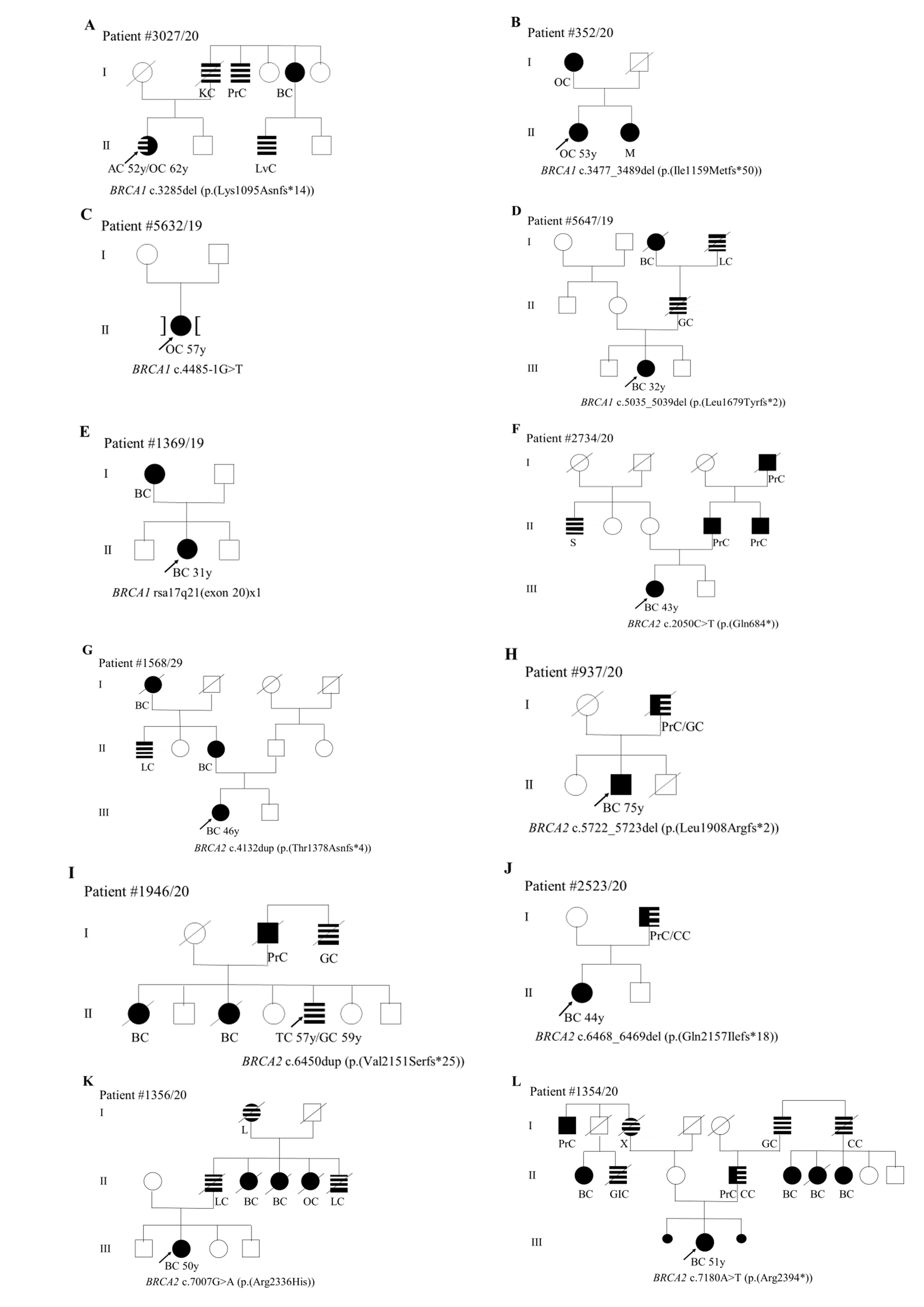

Supplement: Supplementary Figure 2 — Pedigrees of patients carrying pathogenic variants in BRCA1-2. Pedigree of patients #3027/20, 352/20, 5632/20, 5647/19, 2734/20, 1568/20, 937/20, 1946/20, 2523/20, 1356/20, 1354/20, and 1369/19. The gene and their variants are reported below the symbol of each proband. The arrows indicate the proband from each family. The black symbols indicate “HBOC-spectrum phenotypes” while striped symbols indicate “other neoplasms.” Abbreviations as in Figure 1. [file Image_2.jpeg]
