# Supplementary material for: Exome Sequencing in BRCA1-2 Candidate Familias: The Contribution of Other Cancer Susceptibility Genes
Source: Front Oncol. 2021 May 7;11:649435. doi: 10.3389/fonc.2021.649435 (PMC8139251; doi:10.3389/fonc.2021.649435)
Supplement: Supplementary Table 1 — Pathogenic variants found in HBOC genes. HGVS, Human Genome Variation Society (http://www.hgvs.org); ClinVar, Clinical Variation database (https://www.ncbi.nlm.nih.gov/clinvar/); MAF, Minor Allele Frequency; CADD, Combined Annotation Dependent Depletion; NA, non applicable; NR, non reported. [file Table_1.docx]

**TABLE S1:** Pathogenic variants found in HBOC genes

| Patient ID | Gene | Location  (Exon/Intron) | Variant  (HGVS) | Protein  (HGVS) | MAF (gnomAD%) | dpSNP | ClinVar Classification | BRCA Exchange | MaxEntScan | Reference |
| --- | --- | --- | --- | --- | --- | --- | --- | --- | --- | --- |
| 3027/20 | *BRCA1* | 11 | c.3285del | p.(Lys1095Asnfs*14) | NR | rs397509051 | Pathogenic | Pathogenic | NA | Janavičius et al., 2010 [29] |
| 352/20 | *BRCA1* | 11 | c.3477_3480del | p.(Ile1159Metfs*50) | 0.0004 | rs80357781 | Pathogenic | Pathogenic | NA | Vendrell et al., 2018 [30] |
| 5632/19 | *BRCA1* | 14 | c.4485-1G>T | NR | NR | rs80358189 | Pathogenic | Pathogenic | Abolish canonical a splice acceptor site | Manickam et al., 2018 [31] |
| 5647/19 | *BRCA1* | 17 | c.5035_5039del | p.(Leu1679Tyrfs*2) | NR | rs80357623 | Pathogenic | Pathogenic | NA | Laitman et al., 2019 [32] |
| 2734/20 | *BRCA2* | 11 | c.2050C>T | p.(Gln684*) | NR | NR | NR | NR | NA | NR |
| 1568/20 | *BRCA2* | 11 | c.4132dup | p.(Thr1378Asnfs*4) | NR | NR | NR | NR | NA | NR |
| 937/20 | *BRCA2* | 11 | c.5722_5723del | p.(Leu1908Argfs*2) | 0.00040 | rs80359530 | Pathogenic | Pathogenic | NA | Guo et al., 2019 [33] |
| 1946/20 | *BRCA2* | 11 | c.6450dup | p.(Val2151Serfs*25) | NR | rs80359595 | Pathogenic | Pathogenic | NA | Laitman et al., 2019 [32] |
| 2523/20 | *BRCA2* | 11 | c.6468_6469del | p.(Gln2157Ilefs*18) | NR | rs80359596 | Pathogenic | Pathogenic | NA | Laitman et al., 2019 [32] |
| 1356/20 | *BRCA2* | 13 | c.7007G>A | p.(Arg2336His) | NR | rs28897743 | Pathogenic | Pathogenic | NA | Parsons et al., 2019 [34] |
| 1354/20 | *BRCA2* | 14 | c.7180A>T | p.(Arg2394*) | 0.0032 | rs80358946 | Pathogenic | Pathogenic | NA | Palmero et al., 2018 [35] |

Abbreviations: HGVS, Human Genome Variation Society (http://www.hgvs.org); ClinVar, Clinical Variation database (https//www.ncbi.nlm.nih.gov/clinvar/); MAF, Minor Allele Frequency; CADD, Combined Annotation Dependent Depletion; NA, non applicable; NR, non reported.
