# Supplementary material for: Exome Sequencing in BRCA1-2 Candidate Familias: The Contribution of Other Cancer Susceptibility Genes
Source: Front Oncol. 2021 May 7;11:649435. doi: 10.3389/fonc.2021.649435 (PMC8139251; doi:10.3389/fonc.2021.649435)
Supplement: Supplementary Table 2 — VUS variants found in HBOC genes. HGVS, Human Genome Variation Society (http://www.hgvs.org); ClinVar, Clinical Variation database (https://www.ncbi.nlm.nih.gov/clinvar/); MAF, Minor Allele Frequency; CADD, Combined Annotation Dependent Depletion; NA, non applicable; NR, non reported. [file Table_2.docx]

**Table S2:** VUS variants found in HBOC genes

| Patient ID | Gene | Location  (Exon/Intron) | Variant  (HGVS) | Protein  (HGVS) | MAF (gnomAD%) | dpSNP | ClinVar Classification | BRCA Exchange | CADD | MaxEntScan |
| --- | --- | --- | --- | --- | --- | --- | --- | --- | --- | --- |
| 1276/20 | *BRCA1* | 11 | c.1745C>T | p.(Thr582Met) | 0.0032 | rs786202386 | Uncertain significance | NR | 15.7 | NA |
| 197/20 | *BRCA1* | 11 | c.1763_1764delinsTT | p.(Ser588Ile) | NR | rs1555591274 | Uncertain significance | NR | NA | NA |
| 489/20 | *BRCA1* | 11 | c.1881C>G | p.(Val627Val) | 0.0042 | rs80356838 | Uncertain significance | NR | NA | Generation of alternative  5' splice site |
| 1735/20 | *BRCA1* | 11 | c.2431C>T | p.(Pro811Ser) | NR | NR | NR | NR | 10.55 | NA |
| 760/20 | *BRCA1* | 11 | c.3073A>G | p.(Thr1025Ala) | NR | NR | NR | NR | 15.06 | NA |
| 1460/20 | *BRCA1* | 11 | c.3564G>C | p.(Arg1188Ser) | 0.0004 | rs879255484 | Uncertain significance | NR | NA | NA |
| 1499/20 | *BRCA1* | 11 | c.3967C>G | p.(Gln1323Glu) | NR | NR | NR | NR | 15.53 | NA |
| 590/20 | *BRCA2* | 5 | c.476-3C>A | NR | 0.0043 | rs371431745 | NR | NR | 14.13 | Decrease the recognition of the splice acceptor site |
| 1699/20 | *BRCA2* | 10 | c.902A>G | p.(Asp301Gly) | 0.00084 | rs730881508 | Uncertain significance | NR | 14.27 | NA |
| 1346/20 | *BRCA2* | 10 | c.1553C>T | p.(Ala518Val) | NR | NR | NR | NR | 7.249 | NA |
| 510/18 | BRCA2 | 11 | c.6212G>C | p.(Ser2071Thr) | 0.008 | rs80358861 | Uncertain significance | NR | NA | NA |
| 1352/20 | *BRCA2* | 26 | c.9581C>A | p.(Pro3194Gln) | 0.0014 | rs28897760 | Uncertain significance | NR | 23.8 | NA |

Abbreviations: HGVS, Human Genome Variation Society (http://www.hgvs.org); ClinVar, Clinical Variation database (https//www.ncbi.nlm.nih.gov/clinvar/); MAF, Minor Allele Frequency; CADD, Combined Annotation Dependent Depletion; NA, non applicable; NR, non reported.
