# Supplementary material for: Exome Sequencing in BRCA1-2 Candidate Familias: The Contribution of Other Cancer Susceptibility Genes
Source: Front Oncol. 2021 May 7;11:649435. doi: 10.3389/fonc.2021.649435 (PMC8139251; doi:10.3389/fonc.2021.649435)
Supplement: Supplementary Table 3 — VUS found in canonical and non-canonical HBOC genes. HGVS, Human Genome Variation Society (http://www.hgvs.org); ClinVar, Clinical Variation database (https://www.ncbi.nlm.nih.gov/clinvar/); MAF, Minor Allele Frequency; CADD, Combined Annotation Dependent Depletion; NA, non applicable; NR, non reported. [file Table_3.docx]

**Table S3:** VUS found in canonical and non-canonical HBOC genes

| Patient  ID | Gene | Trascript  (hg19) | Location  (Exon/Intron) | Variant  (HGVS) | Protein  (HGVS) | MAF  (gnomAD%) | dpSNP | ClinVar  Classification | CADD |
| --- | --- | --- | --- | --- | --- | --- | --- | --- | --- |
| 2015/20 | *AKT1* | NM_001014432.1 | 15 | c.1394G>A | p.(Arg465His) | 0.0054 | rs113547523 | Uncertain  significance | 23.9 |
| 327/20 | *APC* | NM_000038.4 | 15 | c.2534G>A | p.(Arg845His) | 0.0036 | rs776878597 | Uncertain  significance | 26.6 |
| 3125/20 | *APC* | NM_000038.4 | 15 | c.4918C>T | p.(Arg1640Trp) | 0.0078 | rs373440614 | Uncertain  significance | 22.5 |
| 721/20 | *APC* | NM_000038.4 | 15 | c.7998T>G | p.(Ile2666Met) | NR | NR | Uncertain  significance | 23.4 |
| 1354/20 | *ATM* | NM_00051.3 | 4 | c.202A>G | p.(Ile68Val) | 0.0029 | rs35389822 | Uncertain  significance | 16.88 |
| 1344/20 | *ATM* | NM_00051.3 | 6 | c.655T>C | p.(Cys219Arg) | 0.0008 | rs771685059 | Uncertain  significance | 11.18 |
| 440/20 | *ATM* | NM_00051.3 | 10 | c.1516G>T | p.(Gly506Cys) | 0.0039 | rs587779816 | Uncertain  significance | 25.3 |
| 2691/20 | *ATM* | NM_00051.3 | 17 | c.2587_2589dup | p.(Asp863dup) | NR | NR | NR | NA |
| 2100/20 | *ATM* | NM_00051.3 | 27 | c.4102T>C | p.(Phe1368Leu) | NR | NR | NR | 23.9 |
| 1346/20 | *ATM* | NM_00051.3 | 35 | c.5227A>G | p.(Thr1743Ala) | 0.0004 | rs758924620 | Uncertain  significance | 22.5 |
| 2858/20 | *ATR* | NM_001184.4 | 19 | c.3723C>G | p.(Asn1241Lys) | NR | rs914611293 | NR | 29.1 |
| 2866/20 | *BMPR1A* | NM_004329.2 | 9 | c.731G>A | p.(Arg244Gln) | 0.0008 | rs147971049 | Uncertain  significance | 32 |
| 1572/20 | *CDH1* | NM_004360.3 | 8 | c.1042G>A | p.(Ala348Thr) | NR | NR | Uncertain  significance | 19.59 |
| 399/20 | *CDH1* | NM_004360.3 | 12 | c.1906G>A | p.(Ala636Thr) | 0.0012 | rs876658950 | Uncertain  significance | 21 |
| 3069/20 | *CHEK2* | NM_007194.3 | 3 | c.429C>A | p.(His143Gln) | NR | NR | Uncertain  significance | 24 |
| 1356/20 | *CHEK2* | NM_007194.3 | 6 | c.688G>C | p.(Ala230Pro) | 0.0004 | rs748636216 | Uncertain  significance | 27.6 |
| 2393/20 | *CHEK2* | NM_007194.3 | 11 | c.1169A>C | p.(Tyr390Ser) | 0.0024 | rs200928781 | Uncertain  significance | 28 |
| 691/20 | CHEK2 | NM_007194.3 | 11 | c.1180G>A | p.(Glu394Lys) | 0.002 | rs587780169 | Uncertain  significance | 33 |
| 1354/20 | *ERBB2* | NM_004448.2 | 15 | c.1879C>T | p.(Pro627Ser) | 0.0004 | rs1173337736 | NR | 22.8 |
| 1570/20 | *ERCC1* | NM_202001.2 | 9 | c.799T>C | p.(Ser267Pro) | 0.021 | rs146256515 | Uncertain  significance | 23.5 |
| 1693/20 | *ERCC2* | NM_000400.4 | 14 | c.1348C>T | p.(Arg450Cys) | 0.0008 | rs749815182 | NR | 24.2 |
| 1693/20 | *ERCC2* | NM_000400.4 | 14 | c.1348C>T | p.(Arg450Cys) | 0.0008 | rs749815182 | NR | 24.2 |
| 2866/20 | *FANCD2* | NM_033084.3 | 32 | c.3172A>G | p.(Met1058Val) | 0.0012 | rs9083373952 | NR | 22.8 |
| 2758/20 | *FANCI* | NM_001184.4 | 13 | c.1264G>A | p.(Gly422Arg) | 0.012 | rs146040966 | Uncertain  significance | 31 |
| 325/20 | *FGFR4* | NM_01354984.1 | 7 | c.871G>A | p.(Gly291Ser) | 0.00044 | rs752392002 | NR | 28.1 |
| 1684/20 | *HMMR* | NM_012484.2 | 12 | c.1382G>A | p.(Ser461Asn) | 0.0014 | rs772196576 | NR | 33 |
| 1348/20 | *IGF2R* | NM_000876.3 | 20 | c.2783C>T | p.(Thr928Met) | 0.0088 | rs373268009 | NR | 17.36 |
| 1946/20 | *KRAS* | NM_033360.3 | 5 | c.503T>A | p.(Leu168*) | 0.0016 | rs777244909 | NR | NA |
| 1697/20 | *MAD1L1* | NM_001013836.1 | 18 | c.1891A>G | p.(Lys631Glu) | 0.0004 | rs770694687 | NR | 19.68 |
| 2163/20 | *MLH1* | NM_000249.3 | 2 | c.185A>G | p.(Gln62Arg) | NR | NR | NR | 26.7 |
| 1346/20 | *MLH1* | NM_000249.3 | 14 | c.1564C>T | p.(Arg522Trp) | 0.0018 | rs63751703 | Uncertain  significance | 26.3 |
| 1709/20 | *MLH3* | NM_001040108.1 | 2 | c.3137G>A | p.(Arg1046Gln) | 0.016 | rs201303087 | Uncertain  significance | 14.27 |
| 721/20 | *MSH2* | NM_000251.3 | 1 | c.64T>A | p.(Phe22Ile) | NR | rs1189127007 | Uncertain  significance | 25.5 |
| 553/20 | *MSH2* | NM_000251.3 | 12 | c.1777C>G | p.(Gln593Glu) | NR | rs63750200 | Uncertain  significance | 22.5 |
| 721/20 | *MSH3* | NM_002439.5 | 9 | c.1394A>G | p.(Tyr465Cys) | 0.02 | rs35009542 | Uncertain  significance | 21.4 |
| 2736/20 | *MSH3* | NM_002439.4 | 11 | c.1569G>T | p.(Glu523Asp) | NR | NR | NR | 28 |
| 2803/20 | *MSH6* | NM_000179.2 | 4 | c.663A>C | p.(Glu221Asp) | 0.069 | rs41557217 | Uncertain  significance | 13.94 |
| 2857/19 | *MTOR* | NM_004958.3 | 46 | c.6401G>A | p.(Arg2134Gln) | 0.0008 | rs755115241 | NR | 29.2 |
| 939/20 | *MTOR* | NM_004958.3 | 46 | c.6454C>T | p.(Arg2152Cys) | NR | rs752458445 | NR | 23.6 |
| 3069/20 | MUTYH | NM_001128425.1 | 12 | c.1103G>A | p.(Arg368Lys) | 0.003 | rs1060501340 | Uncertain  significance | 26.7 |
| 190/20 | *NQO1* | NM_000903.2 | 6 | c.601C>T | p.(Arg201*) | 0.0012 | rs754187281 | NR | 12.65 |
| 1268/20 | *PALB2* | NM_024675.3 | 4 | c.764A>G | p.(Asp255Gly) | 0.004 | rs776753788 | Uncertain  significance | 25.8 |
| 3173/20 | *PALB2* | NM_024675.3 | 4 | c.1408A>G | p.(Thr470Ala) | 0.0012 | rs150636811 | Uncertain  significance | 0.008 |
| 2134/20 | *PALB2* | NM_024675.3 | 4 | c.1882_1890del | p.(Lys628_Cys630del) | 0.0032 | rs587778583 | Uncertain  significance | NA |
| 3025/20 | *PALB2* | NM_024675.3 | 5 | c.2106A>G | p.(Ile702Met) | 0.0011 | rs730881886 | Uncertain  significance | 0.01 |
| 2477/20 | *PALB2* | NM_024675.3 | 11 | c.3128G>C | p.(Gly1043Ala) | 0.0016 | rs377713277 | Uncertain  significance | 29.7 |
| 1735/20 | *PALB2* | NM_024675.3 | 12 | c.3296C>G | p.(Thr1099Arg) | 0.006 | rs142132127 | Uncertain  significance | 27.7 |
| 2335/20 | *POLD1* | NM_001308632.1 | 1 | c.521G>A | p.(Arg174Gln) | 0.0085 | rs141976385 | Uncertain  significance | 16.06 |
| 2758/20 | *POLE* | NM_006231.2 | 3 | c.218A>G | p.(Asp73Gly) | 0.0004 | rs1060500786 | Uncertain  significance | 27 |
| 1693/20 | *POLE* | NM_006231.2 | 13 | c.1354C>A | p.(Phe452Thr) | NR | rs1555228573 | Uncertain  significance | 24.4 |
| 1348/20 | *POLE* | NM_006231.2 | 24 | c.2770C>T | p.(Arg924Cys) | 0.0025 | rs369751686 | Uncertain  significance | 23.9 |
| 2858/20 | *POLE* | NM_006231.2 | 38 | c.5095G>T | p.(Ala1688Ser) | NR | NR | NR | 22.9 |
| 5130/19 | *PTEN* | NM_000314.4 | 8 | c.862G>A | p.(Glu288Lys) | NR | NR | Uncertain  significance | 23.5 |
| 553/20 | *RAD50* | NM_005732.3 | 11 | c.1663A>G | p.(Ile555Val) | 0.0082 | rs201120953 | Uncertain  significance | 21.1 |
| 1164/20 | *RAD51B* | NM_001321821.1 | 8 | c.767C>T | p.(Thr256Met) | 0.0012 | rs373104809 | NR | 28.7 |
| 1268/20 | *RAD51D* | NM_001142571.1 | 3 | c.322C>T | p.(Arg108Cys) | 0.0056 | rs142387263 | Uncertain  significance | 0.002 |
| 3216/20 | *RAD51D* | NM_001142571.1 | 4 | c.330T>A | p.(Asp110Glu) | NR | NR | Uncertain  significance | 21.8 |
| 880/20 | *RAD54L* | NM_001142548.1 | 10 | c.1093C>T | p.(Arg365*) | 0.0016 | rs559500678 | NR | 36 |
| 1215/20 | *RAD54L* | NM_001142548.1 | 14 | c.1517C>T | p.(Ala506Val) | 0.0018 | rs780047827 | NR | 29.3 |
| 1301/20 | *RAD54L* | NM_001142548.1 | 14 | c.1592A>C | p.(Lys531Thr) | 0.00041 | NR | NR | 24.4 |
| 2693/20 | *RAD54L* | NM_001142548.1 | 18 | c.2095T>C | p.(Cys699Arg) | NR | rs75696652 | NR | 29 |
| 2891/20 | *RET* | NM_020975.4 | 7 | c.1291C>G | p.(Gln431Glu) | NR | NR | NR | 14.01 |
| 399/20 | *RET* | NM_020975.4 | 20 | c.3233C>T | p.(Thr1078Met) | 0.0024 | rs762952212 | Uncertain  significance | 25.8 |
| 1699/20 | *SDHB* | NM_003000.2 | 6 | c.544G>A | p.(Gly182Arg) | 0.0014 | rs201928318 | Uncertain  significance | 32 |
| 2423/20 | *SLC22A18* | NM_002555.5 | 11 | c.1165C>T | p.(Arg389Cys) | 0.33 | rs141445711 | NR | 23.5 |
| 2897/20 | *SLC22A18* | NM_002555.5 | 4 | c.286T>G | p.(Phe96Val) | 0.0072 | rs779619751 | NR | 21.4 |
| 1697/20 | *SLC22A18* | NM_002555.5 | 7 | c.664C>T | p.(Arg222Trp) | 0.0029 | rs200758237 | NR | 4.666 |
| 832/20 | *STK11* | NM_000455.4 | 4 | c.539G>T | p.(Gly180Val) | NR | NR | NR | 25.2 |
| 399/20 | *STK11* | NM_000455.4 | 9 | c.1225C>T | p.(Arg409Trp) | 0.0087 | rs368466538 | Uncertain  significance | 21.9 |
| 1684/20 | *TFF1* | NM_003225.2 | 2 | c.188G>T | p.(Arg63Leu) | 0.0081 | rs141090896 | NR | 0.2 |
| 1568/20 | *TP53* | NM_000564.4 | 5 | c.431A>T | p.(Gln144Leu) | NR | rs786203071 | Uncertain  significance | 23.7 |
| 2245/20 | *TP53* | NM_000564.4 | 8 | c.845G>T | p.(Arg282Leu) | NR | rs730882008 | Uncertain  significance | 27.7 |
| 1920/20 | *TP53* | NM_000564.4 | 8 | c.859G>A | p.(Glu287Lys) | 0.0004 | rs587782006 | Uncertain  significance | 22.9 |
| 2006/20 | *XRCC3* | NM_001100119.1 | 10 | c.844C>G | p.(Pro282Ala) | NR | NR | NR | 23.4 |

Abbreviations: HGVS, Human Genome Variation Society (http://www.hgvs.org); ClinVar, Clinical Variation database (https//www.ncbi.nlm.nih.gov/clinvar/); MAF, Minor Allele Frequency; CADD, Combined Annotation Dependent Depletion; NA, non applicable; NR, non reported.
