# Supplementary material for: Exome Sequencing in BRCA1-2 Candidate Familias: The Contribution of Other Cancer Susceptibility Genes
Source: Front Oncol. 2021 May 7;11:649435. doi: 10.3389/fonc.2021.649435 (PMC8139251; doi:10.3389/fonc.2021.649435)
Supplement: Supplementary Table 4 — Comparison of variant frequencies with an internal biobank of 1022 samples (median range 34-95 years) without any history of tumor and analysed by ES. HGVS, Human Genome Variation Society (http://www.hgvs.org). [file Table_4.docx]

**Table S4**: Comparison of variant frequencies with an internal biobank of 1022 samples (median range 34-95 years) without any history of tumor and analysed by WES.

| Gene | Trascript  (hg19) | Location (Exon/Intron) | Variant (HGVS) | Protein  (HGVS) | Variant frequencies |
| --- | --- | --- | --- | --- | --- |
| *DPYD* | NM_000110.3 | 15 | c.1905+1G>A | NA | 0,4%  (4/1022) |
| *ERBB3* | NM_001982.3 | 3 | c.277G>T | p.(Glu93*) | 0%  (0/1022) |
| *ERCC2* | NM_000400.3 | 22 | c.2164C>T | p.(Arg722Trp) | 0%  (0/1022) |
| *MUTYH* | NM_001128425.1 | 5 | c.421del | p.(Gln141Argfs*5) | 0%  (0/1022) |
| *MUTYH* | NM_001128425.1 | 7 | c.536A>G | p.(Tyr179Cys) | 0,6%  (6/1022) |
| *MUTYH* | NM_001128425.1 | 9 | c.733C>T | p.(Arg245Cys) | 0%  ( 0/1022) |
| *MUTYH* | NM_001128425.1 | 10 | c.933+3A>C | NA | 0,2%  (2/1022) |
| *NQO2* | NM_000904.4 | 5 | c.418-2A>G | NA | 0%  (0/1022) |
| *NTHL1* | NM_02528.5 | 2 | c.268C>T | p.(Gln90*) | 0,4%  (4/1022) |
| *PARK2* | NM_004562.2 | 2 | c.125G>C | p.(Arg42Pro) | 0%  (0/1022) |
| *RAD54L* | NM_001142548.1 | 10 | c.1093C>T | p.(Arg365*) | 0%  (0/1022) |
| *RNASEL* | NM_021133.3 | 2 | c.793G>T | p.(Glu265*) | 0,9%  (9/1022) |

Abbreviations: HGVS, Human Genome Variation Society (http://www.hgvs.org).
